# A hybrid learning-based stochastic noise eliminating method with attention-Conv-LSTM network for low-cost MEMS gyroscope

**DOI:** 10.3389/fnbot.2022.993936

**Published:** 2022-12-15

**Authors:** Yaohua Liu, Jinqiang Cui, Wei Liang

**Affiliations:** ^1^School of Nano-Tech and Nano-Bionics, University of Science and Technology of China, Hefei, China; ^2^Institute of Nano-Tech and Nano-Bionics, Chinese Academy of Sciences, Suzhou, China; ^3^Department of Mathematics and Theories, Peng Cheng Laboratory, Shenzhen, China

**Keywords:** MEMS IMU, deep learning, noise reduction, inertial navigation, random noise

## Abstract

Low-cost inertial measurement units (IMUs) based on microelectromechanical system (MEMS) have been widely used in self-localization for autonomous robots due to their small size and low power consumption. However, the low-cost MEMS IMUs often suffer from complex, non-linear, time-varying noise and errors. In order to improve the low-cost MEMS IMU gyroscope performance, a data-driven denoising method is proposed in this paper to reduce stochastic errors. Specifically, an attention-based learning architecture of convolutional neural network (CNN) and long short-term memory (LSTM) is employed to extract the local features and learn the temporal correlation from the MEMS IMU gyroscope raw signals. The attention mechanism is appropriately designed to distinguish the importance of the features at different times by automatically assigning different weights. Numerical real field, datasets and ablation experiments are performed to evaluate the effectiveness of the proposed algorithm. Compared to the raw gyroscope data, the experimental results demonstrate that the average errors of bias instability and angle random walk are reduced by 57.1 and 66.7%.

## 1. Introduction

Recently, with the development of the microelectromechanical system (MEMS) and artificial intelligence (AI), the low-cost MEMS inertial measurement units (IMUs) are essential for many applications, such as unmanned aerial vehicles, autonomous driving, mobile robots, etc. IMUs consist of gyroscopes that measure angular velocities and accelerometers that measure the accelerations of moving vehicles. The IMUs can provide the entire attitude, velocity, and position information through the integral operation. However, the measurement errors will accumulate over time due to the bias error instability and stochastic noise in raw IMU data. Specifically, the position error of inertial navigation diverges with the second power of accelerometer bias drift and time, and diverges with the third power of gyroscope bias drift and time. Therefore, modeling or denoising the low-cost MEMS IMUs is crucial to improving the inertial navigation system (INS) performance.

In inertial navigation, the errors contained in the MEMS IMU raw signals can be divided into two parts: deterministic and stochastic errors. The deterministic error part mainly includes the scale factor error and axes misalignment error, which can be calibrated or quantified by equipment such as a 3-axis turntable. While the stochastic errors consist of bias error and noise, they are hard to calibrate due to their time-varying characteristic. Thus, the stochastic errors are also a vital issue of the INS errors divergence. In order to identify and model the stochastic error, researchers have proposed many representative denoising techniques for MEMS IMUs, which can be inclusively divided into conventional signal processing methods and recent learning-based methods. Auto Regressive Moving Average Method (ARMA) (Song et al., 2018), Allan Variance (Zhang et al., 2018), Kalman Filter (Zhang et al., 2016), and Wavelet Transformation (WT) (Yuan et al., 2015) are the representative signal processing methods. The ARMA method is mainly used to analyze and study a group of stochastic data arranged in sequence. It establishes mathematical models of various orders according to different error sequences. However, this method cannot identify stochastic errors one by one, and it is difficult to distinguish the error sources of stochastic errors. Allan Variance can identify various stochastic errors and separate them into five parts: quantization noise, angular random walk, bias instability, rate random walk, and rate ramp. Thus, the advantage of the Allan Variance is that it can draw a double logarithmic curve to connect the time and frequency domains and visually observe the stochastic errors quantitatively. When the amount of data is large enough, the drawn double logarithmic curve is more intuitive and straightforward (El-Sheimy et al., 2007). Kalman Filter is an efficient linear quadratic estimator which can estimate gyroscope output angular velocity *via* a series of observed measurements with noise (Cai et al., 2018). Since the natural MEMS IMU error system is usually too complex to build an accurate mathematics model, the Kalman filter has a poor performance in estimation accuracy. Among the signal processing methods, the Wavelet Transform method is currently most popular for reducing the high-frequency part of the gyroscope error. However, it is hard to remove the low-frequency errors (Ding et al., 2021). The the signal processing algorithms can only reduce part of the MEMS gyroscope stochastic error, and the unsatisfactory suppression of the stochastic errors will cause the failure of inertial navigation in a short time.

Other learning-based approaches are proposed to improve the traditional statistical algorithms, such as support vector machine (SVM) and neural networks, all of which obtain better denoising results than conventional signal processing methods (Leung et al., 2001; Shiau et al., 2011; Bhatt et al., 2012). In Zhang and Yang (2012), the SVM is utilized to model and compensate for the angular rate error of MEMS gyroscope MG31-300, which indicates that the SVM model has high precision and good generalization ability. A basis function neural network is adopted to predict the noisy chaotic time series due to its non-linear, adaptive, and self-learning characteristics (Leung et al., 2001). Gonzalez and Catania (2019) proposed a rigorous analysis of the viability of the Time Delayed Multiple Linear regression techniques for reducing white noise in the MEMS IMU. Their advantages rely on their ability to identify complex patterns by learning high-level data features. However, almost all of the above methods are based on a static model, which models only the current and past one-step angular velocity information and can not store more past gyroscope dynamic information. It is known that gyroscope data is time serial data in which the history error will affect the current measurement value.

In recent years, deep learning has achieved outstanding performances in computer vision (Han et al., 2020) and natural language processing (Koroteev, 2021) due to their powerful non-linear modeling and feature representation. Some researchers have introduced deep learning into the inertial odometer, such as OriNet (Esfahani et al., 2019), IONet (Chen et al., 2018), TLIO (Liu et al., 2020), all of which obtained excellent localization performance than traditional methods. However, the use of deep learning technology to reduce MEMS IMU stochastic noise has just begun, and the published research results are still rare. In Jiang et al. (2018a), an recurrent neural network (RNN) variant simple recurrent unit (SRU-RNN) is employed in MEMS gyroscope raw signal denoising. The Allan variance tool is also used to compute the major error factors, i.e., quantization noise, angle random walk, and bias instability. However, RNN performs poorly in long sequences due to gradient disappearance and gradient explosion. To solve such problem, long short-term memory (LSTM) has been proposed (Graves et al., 2005; Sherstinsky, 2020), which can be used to denoise the MEMS gyroscope based on the current and previous angular velocities. In Jiang et al. (2018b), the LSTM is employed to filter the MEMS gyroscope outputs by treating the signals as time series. The results indicated that the denoising scheme effectively improves MEMS gyroscope accuracy. To further explore the effect of LSTM in denoising the MEMS gyroscope, some hybrid deep recurrent neural networks, including LSTM and gated recurrent unit (GRU), are evaluated for MEMS IMU with static and dynamic conditions (Han et al., 2021). The LSTM is also combined with the Kalman filter to estimate and compensate for the random drift of the MEMS gyroscope in real-time (Li et al., 2021; Zhu et al., 2021). It is noted that the RNN can learn the temporal correlation from the useful signals of the original data, but it cannot learn from the noisy components (Shiau et al., 2011). Thus, RNNs have a poor ability to extract the local features of MEMS gyroscope. To solve the problems, a convolutional neural network (CNN) is applied to reduce the attitude angle errors and achieve better denoising performances (Brossard et al., 2020). However, we focus on eliminating the stochastic noise in raw MEMS gyroscope data, rather than calibrating IMU error by reducing the attitude angle error.

As already discussed above, it can be seen that most eliminating MEMS gyroscope stochastic noise works (90%) are based on the RNNs; significantly, only one hybrid model with LSTM and GRU. None of the above methods can simultaneously extract the local features of the MEMS gyroscope and learn the long-range dependence. In addition, they can not explore different levels of the importance of gyroscope sequences at different times.

Therefore, this paper aims to develop a hybrid MEMS gyroscope denoising scheme based on Attention-CNN-LSTM (ACL) to eliminate the stochastic noise for angular velocity. Although there are similar hybrid models in other fields, such as stock prediction, we focus on MEMS gyroscope stochastic noise reduction, and there is no research on the hybrid denoising model so far. Specially, a one-dimensional CNN is adopted in the proposed ACL to extract local MEMS gyroscope features. The features are fed to the LSTM layer to mine the temporal features further and learn the long-term historical dependence. In order to improve computing efficiency, an attention mechanism is applied to distinguish the importance of MEMS gyroscope sequences at different times. The contributions of the paper are summarized as follows:

1. We develop a hybrid denoising model based on Conv-LSTM networks to capture the spatial-temporal feature of the MEMS gyroscope sequence. Unlike the existing RNN-based method for denoising gyroscopes, Conv-LSTM can capture the sectional features and learn long-range dependencies simultaneously, which is more efficient for mining the inherent characteristic of the gyroscope sequence.
2. We embed an attention mechanism for the Conv-LSTM model to automatically allocate different attention weights to a gyroscope sequence at different times, which can further improve the efficiency of the Conv-LSTM model.
3. A series of experiments are performed to verify the effectiveness of the proposed method. The experimental results demonstrate that the proposed model performs better than other gyroscope denoising methods.

The remainder of the paper is organized as follows. Section 2 explains the mathematical model of low-cost MEMS IMU in detail. Section 3 describes the process of establishing a denoising model based on ACL. Real field, datasets and ablation experiments and results analysis are discussed in Section 4. The conclusion is provided in Section 5.

## 2. The mathematical models of low-cost MEMS IMU

Low-cost MEMS IMUs are prone to various errors, which get more complex as the sensor price decreases. The errors limit the accuracy to which the observables can be measured. In this section, the output models of the MEMS IMUs are presented to analyze their error characteristics.

### 2.1. The errors of the low-cost MEMS IMUs

MEMS IMU contains two orthogonal sensor triads, one with three accelerometers and the other with three gyroscopes. Accelerometers measure linear motion in three orthogonal directions, whereas gyroscopes measure angular motion in three orthogonal directions. However, owing to the limitation of current MEMS manufacturing technology, the output of the MEMS IMU is affected by many error sources.

The general terms of repeatability, stability, and drift are usually considered to assess a MEMS IMU sensor for a particular application. The repeatability term represents the ability of a MEMS IMU to provide the same output for repeated applications of the same input. It refers to the maximum variation between repeated measurements in the same conditions over multiple runs. The stability term illustrates the ability of a MEMS IMU to provide the same output when measuring a constant input over a while. The term drift is often used to describe the change in the MEMS IMU measurement when there is no change in the input. Especially the MEMS IMU errors can be classified into two broad categories of deterministic and stochastic errors. Deterministic errors mainly include systematic bias offset, scale factor error, non-linearity, non-orthogonality error, and misalignment error. Most of the deterministic errors can only be found in dynamic environments, and can be compensated by laboratory calibration process. Low-cost MEMS IMUs suffer from various stochastic errors, which are usually modeled stochastically to mitigate their effects. In general, the stochastic errors of Low-cost MEMS IMU can be divided into run-to-run bias offset, bias drift, scale factor instability, and white noise. Any above stochastic errors will cause the navigation results (attitude, velocity, and position) to diverge rapidly in the inertial navigation system. Therefore, it is fundamental to suppress the stochastic errors of the low-cost MEMS IMUs.

The initial error of IMU is relatively tiny, but as time goes on, the position and speed position calculated by the inertial navigation algorithm will become larger and larger. The position of inertial navigation can be expressed as follows,


(1) $$\begin{array}{l} \delta r_{N}=\delta r_{N,0}+\delta v_{N,0}\cdot t+\frac{1}{2}\left(g\cdot \delta \theta_{0}+b_{aN}\right)t^{2}+\frac{1}{6}\left(g\cdot b_{gE}\right)t^{3} \end{array}$$

where δ*r*_*N*_ is north position error, δ*r*_*N*, 0_, δ*v*_*N*, 0_, and δθ_0_ represent north position error, velocity error and yaw angle error at initial time, respectively. *b*_*aN*_ and *b*_*gE*_ are the bias error of the accelerometer in the north direction and gyroscope in the east direction. *g* is local gravity, and *t* is inertial navigation time. It can be seen that the bias drift of the accelerometer will cause position error to diverge with the second power of time, and the bias drift of the gyroscope will cause position error to diverge with the third power of time. If the MEMS IMU is not denoised well, the position information calculated by the MEMS IMU will not be used for navigation.

### 2.2. The output model base on low-cost MEMS IMUs

In the field of inertial navigation, the output model based on low-cost MEMS IMUs includes the angular rate model and the specific force model, i.e., the measurements of the gyroscope and accelerometer, respectively. Measurements of angular rate can be expressed as follows:


(2) $$\begin{array}{l} {\tilde{\omega }}_{ib}^{b}=\omega_{ib}^{b}+b_{g}+S_{g}\omega_{ib}^{b}+N_{g}\omega_{ib}^{b}+\varepsilon_{g} \end{array}$$

where $\omega_{ib}^{b}$ is the real values of the angular velocity in the body frame *b* relative to the inertial frame *i*, and ${\tilde{\omega }}_{ib}^{b}$ is the output values of the gyroscope. Furthermore, *b*_*g*_, ε_*g*_, *S*_*g*_, and *N*_*g*_ are the gyroscope instrument bias vector, noise vector, scale factor matrix and non-orthogonality matrix, respectively. The bias vector is defined as the gyroscope's output when there is zero input. The noise vector is white noise, which can be caused by power sources but can also be intrinsic to semiconductor devices. The scale factor matrix reflects the deviation of the input-output gradient from unity. As the name suggests, non-orthogonality errors occur when any of the axes of the gyroscope triad depart from mutual orthogonality. The matrices *N*_*g*_ and *S*_*g*_ are given as,


(3) $$\begin{array}{l} N_{g}=\left[\begin{array}{ccc} 1 & \theta_{g,xy} & \theta_{g,xz}\\ \theta_{g,yx} & 1 & \theta_{g,yz}\\ \theta_{g,zx} & \theta_{g,zy} & 1 \end{array}\right],S_{g}=\left[\begin{array}{ccc} s_{g,x} & 0 & 0\\ 0 & s_{g,y} & 0\\ 0 & 0 & s_{g,z} \end{array}\right] \end{array}$$

where θ_*g*, ._ are the small angles defining the misalignments between the different gyroscope axes and *s*_*g*, ._ are the scale factors for the three gyroscopes.

The attitude angular increment is obtained by integrating the measured value of the gyroscope, namely,


(4) $$\begin{array}{l} R\left(t\right)=R\left(t-1\right)\exp\left(\theta_{t}\right)\\ \theta_{t}={\tilde{\omega }}_{ib}^{b}\left(t\right)dt\\ \exp\left(\theta_{t}\right)=I+\frac{\sin\theta_{t}}{\theta_{t}}\left[\theta_{t}\times \right]+\frac{1-\cos\theta_{t}}{\theta_{t}^{2}}{\left[\theta_{t}\times \right]}^{2} \end{array}$$

where ${\tilde{\omega }}_{ib}^{b}\left(t\right)$ is the output of the gyroscope and is also the angular velocity of the body frame *b* relative to the inertial frame *i*, *R*(*t*) is the rotation matrix of the body frame *b* relative to the inertial frame *i*, [θ_*t*_×] is the antisymmetric matrix of θ_*t*_, θ_*t*_ is attitude angles.

From Equation (2), *S*_*g*_ and *N*_*g*_ can be reduced by the calibration processing with a turntable. *b*_*g*_ and ε_*g*_ are hard to estimated by traditional method due to their time-varying characteristic. If the errors cannot be reduced, the errors will be transferred to the rotation matrix and they will accumulate over time according to Equation (2). Thus, our goal is to establish a denoising model based on deep learning to reduce *b*_*g*_ and ε_*g*_. In other words, we use the deep learning model to denoise the gyroscope, reducing the errors of *b*_*g*_ and ε_*g*_, so that the gyroscope measurements ${\tilde{\omega }}_{ib}^{b}$ are closer to the true value $\omega_{ib}^{b}$, and the attitude angles θ_*t*_ can be estimated more accurately through Equation (2).

The output error model of the accelerometer is similar to those which characterize the gyroscope accuracy bias uncertainty, scale factor stability, and random noise. Measurement of the specific force can be modeled by the observation equation,


(5) $$\begin{array}{l} {\tilde{f}}^{b}=f^{b}+b_{a}+S_{1}f+S_{2}f^{2}+N_{a}f+\delta g+\varepsilon_{a} \end{array}$$

where ${\tilde{f}}^{b}$, *f*^*b*^, *b*_*a*_, δ*g*, and ε_*a*_ are the vectors of the accelerometer measurement, the true specific force, the accelerometer instrument bias, the anomalous gravity and noise, respectively. Similar to the gyroscope, *S*_1_, *S*_2_, and *N*_*a*_ are the error matrices of linear scale factor, non-linear scale factor and non-orthogonality. The matrices *N*_*a*_, *S*_1_, and *S*_2_ are defined as follows,


(6) $$\begin{array}{l} N_{a}=\left[\begin{array}{ccc} 1 & \theta_{a,xy} & \theta_{a,xz}\\ \theta_{a,yx} & 1 & \theta_{a,yz}\\ \theta_{a,zx} & \theta_{a,zy} & 1 \end{array}\right],S_{1}=\left[\begin{array}{ccc} s_{1,x} & 0 & 0\\ 0 & s_{1,y} & 0\\ 0 & 0 & s_{1,z} \end{array}\right],\\ S_{2}=\left[\begin{array}{ccc} s_{2,x} & 0 & 0\\ 0 & s_{2,y} & 0\\ 0 & 0 & s_{2,z} \end{array}\right] \end{array}$$

where θ_*a*, *_ are the small angles defining the misalignments between the different accelerometer axes and *s* are the scale factors for the three accelerometers.

## 3. MEMS IMU stochastic errors reduction method based on deep learning

In order to improve the accuracy of the low-cost MEMS IMUs, a hybrid deep learning model with attention-based CNN-LSTM networks is proposed to reduce stochastic errors. In this section, the network architecture is illustrated and the principles of CNN, LSTM and attention mechanism are also introduced.

### 3.1. Network architecture

**Figure 1:**
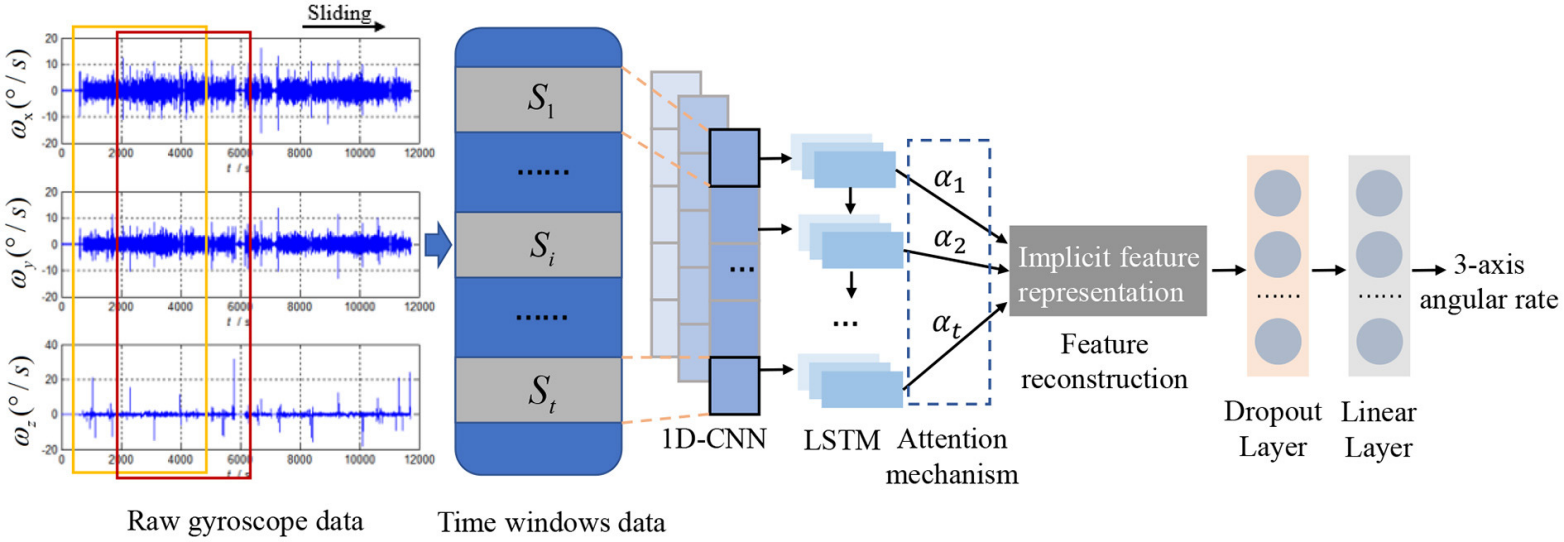
The architecture of the prediction model.

As illustrated in Figure 1, the deep learning model of denoising low-cost MEMS IMU, namely ACL, mainly consists of 1D-CNN layers, LSTM layers, and an attention mechanism. Since the error characteristics of MEMS gyroscope and accelerometer are similar, and gyroscope is essential for inertial navigation, we will take gyroscope data as an example to analyze the noise reduction process based on the proposed ACL model. The raw angular rate signals from the MEMS gyroscope sensors can be observed within the different time windows. Moreover, the *i*_*th*_ observed data *S*_*i*_ is fed into the 1D-CNN layers, which act as feature extractors to automatically obtain the local features and provide abstract representations of the input sensor data in the feature maps. So the noise reduction problem can be formulated as follows,


(7) $$\begin{array}{l} Denoised\text{\_}Gyro=ACL-NN\left(S_{1},S_{2},...,S_{k}\right) \end{array}$$

LSTM layers can further learn the long-term historical dependence from the results of the previous convolution output. Meanwhile, an attention mechanism is designed to explore different levels of the importance of gyroscope sequences at different times. A dropout layer is also applied to avoid overfitting. A linear layer is added to transform high dimension data as the output data dimension shape to predict low noise angular rate. Each module will be described in detail in the following subsections.

### 3.2. One dimensional convolutional neural network (1D-CNN)

1D-CNN is widely used in time series analysis, audio signal data with fixed length periods, natural language processing, etc. The angular velocities and accelerations of the vehicles measured by MEMS IMUs can be regarded as a kind of time-series sequence. For example, the gyroscope sequences of the *i*_*th*_ time window can be expressed,


(8) $$\begin{array}{l} S_{i}=\left[x_{1},x_{2},...,x_{l}\right] \end{array}$$

where *l* is window size and *x*_*t*_ represents the raw angular velocities from the gyroscope at time *t*.

**Figure 2:**
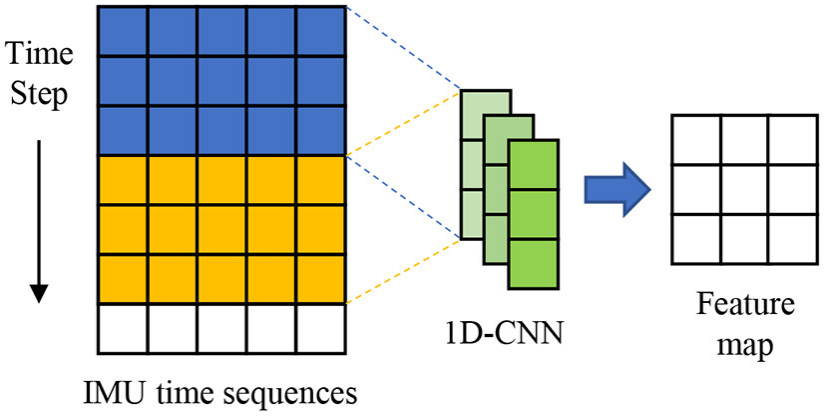
Illustration of the 1D convolution operation.

The 1D-CNN is used to extract local error features from raw MEMS IMU data in our proposed method. The specific convolution operation is,


(9) $$\begin{array}{l} c_{k}=\text{Re}LU\left(\omega_{k}*x+\text{b}\right) \end{array}$$

where *c*_*k*_ is the output feature map of the *k*_*t*_*h* kernel, ω_*k*_ and *b* are weight and deviation parameters. As shown in Figure 2, the convolution operator slides along the time direction and outputs the feature map. Since the 3-axis gyroscope data is fed to 1D-CNN in our model, the number of input channel is set 3. The gyroscope records the angular velocity of the carrier at each time, so the length of the gyroscope measurement sequence cannot be changed. To ensure that the input sequence and output sequence of the gyroscopes are the same length after 1D-CNN operation and reduce the model parameters, the convolution kernel size and the layer of 1D-CNN are set to 1. In order to improve the representation ability of extracted features, the output channel size of 1D-CNN is 256.

### 3.3. Long short term memory (LSTM)

RNN is a popular branch of the deep learning method, where the connections among nodes can form a directed graph along a sequence. Unlike feedforward neural networks, RNNs can use their internal state (memory) to process sequences of inputs. However, it has a vanishing gradient problem that is unable to find an appropriate gradient in long-term memory (Gers et al., 2000; Sutskever et al., 2014).

**Figure 3:**
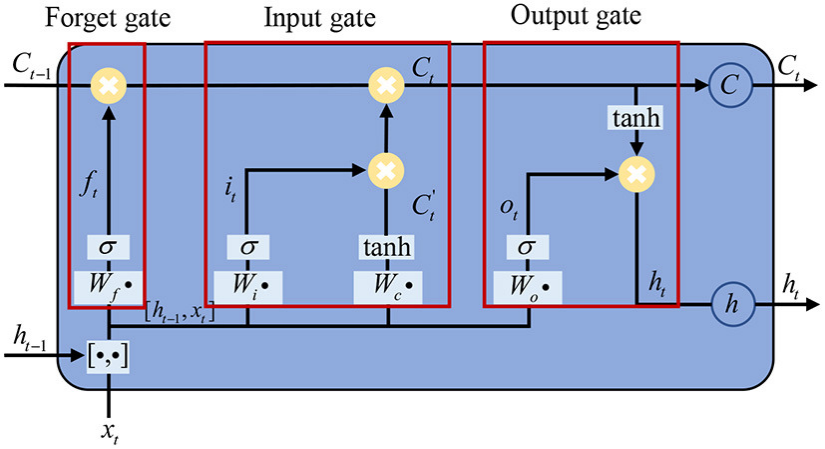
Illustration of the LSTM network.

An RNN composed of LSTM units is often called an LSTM network, which contains a cell, an input gate, an output gate, and a forgetting gate to avoid the vanishing gradient problem. An LSTM memory unit is shown in Figure 3. LSTM uses two gates to control the contents of the unit state *C*. One is the forgetting gate, which determines how much of the cell state in the previous moment *C*_*t*−1_ is retained in the current cell state *C*_*t*_. The other one is the input gate, which determines the level of input of the current network *X*_*t*_ is saved to the cell state *C*_*t*_. The LSTM NN uses the output gate to control the level of the unit state *C*_*t*_ sent to the current output *h*_*t*_ (Sak et al., 2014). The current input cell status ${\tilde{C}}_{t}$ can be calculated based on the previous output. The final LSTM output is determined by both the output gate and the unit state.

In our model, the LSTM input channel size is the same as the previous 1D-CNN output channel size, i.e., 256, and the output channel size is 128.

### 3.4. Attention mechanism

The attention mechanism is a technique that mimics human cognitive attention by selectively ignoring part of the unimportant information and focusing on specific objects. It lays the groundwork for variants of subsequent attention mechanisms and has been successfully used in computer vision, recommendation systems, and translation (Bahdanau et al., 2014). In the context of neural networks, the attention mechanism can be regarded as a weight matrix. In other words, each input data have a corresponding weight value by assigning the attention degree, and the stronger the attention, the greater the weight.

As is known, the time sequence data of the MEMS gyroscope contain more complex temporal information. The error features information of the MEMS gyroscope computed by the LSTM at different times may influence the angular velocities differently. For example, the initial error at a time window will accumulate over time and have a greater impact than the error at the end of the time window. However, the standard LSTM cannot deal with the different important parts of the gyroscope sequence well. Therefore, soft attention (Zhao et al., 2020) is adopted to automatically distinguish different levels of importance of the error features at different times. The attention mechanism can be expressed as,


(10) $$\begin{array}{l} \alpha_{i}=\frac{\exp\left(s_{i}\right)}{\sum_{i=1}^{t-1}\exp\left(s_{i}\right)} \end{array}$$

where α_*i*_ represents the importance of the *i*_*t*_*h* time window for MEMS gyroscope sequence prediction, and the score *s*_*i*_ is the attention weight.

## 4. Experimental results and analysis

The real field, dataset and ablation tests are performed in this section to evaluate the proposed algorithm. Allan variance is used to quantitatively analyze the stochastic noise reduction effects.

### 4.1. Real field tests

In order to verify the performance of our method, a popular low-cost MEMS IMU AHRS380SA-200 manufactured by ACEINNA company is employed in this study. The IMU is composed of 3-orthogonal gyroscopes and 3-orthogonal accelerometers. As listed in Table 1, the full measurement range, maximum bias instability and angle random walk of the AHRS380SA-200 are ±180°/*s*, 10°/*h* and $0.75^{•}/\sqrt{hr}$.

**Table 1:** The specifications of the AHRS380SA-200.

		Range (**°**/***s***)	± 180	
	Gyroscope	Bias instability (**°**/***hr***)	< 10	
		Angular random walk (${}^{•}/\sqrt{hr}$)	< 0.75	
		Range (***g***)	± 4	
	Accelerometer	Bias instability (***mg***)	< 0.02	
		Velocity random walk ($m/s/\sqrt{hr}$)	< 0.05	
	Physical	Size (***mm***)	41*48*22	
		Weight (***gm***)	< 30	
		Output data rate (***Hz***)	2 to 100	
	Electrical	Input voltage (***VDC***)	9–32	
		Power consumption (***mW***)	< 350	

During the raw signal collecting, the AHRS380SA-200 is placed on the table statically, and the sampling frequency is set to 100 Hz at room temperature. A computer-installed data acquisition software retrieved the raw signals *via* a MOXA USB to RS-232 data conversion cable. The Pytorch 1.8 is used as the deep learning framework tool, and the computer used in the experiment is configured as Intel Corei7-6700 3.4 GHz, 16GB RAM, RTX2080ti GPU. Two hours of gyroscope output data is used to train the model. In contrast, the same raw data length is adopted to evaluate the model's performance and tune the model parameters. The Allan Variance method is selected to analyze and describe the composition of the gyroscope noise contained in the raw output signals, which is a time-domain analysis technique originally designed for characterizing noise and stability in clock systems (Woodman, 2007). For LSTM networks, the sequence length can determine how much context information is sent to the model each time. Considering the IMU sampling rate, a window size of 100 is applied to the IMU sequence data to reduce memory. In order to reduce the risk of overfitting and accelerate the training speed, the Adam optimizer (Kingma and Ba, 2014) with cosines warning restart scheduler (Loshchilov and Hutter, 2016) is adopted. To achieve the best performance of the ACL in the experiment, the parameters of the model are fully tuned. The hyperparameters are list in Table 2, where the learning rate is initialized at 0.0001, the batch size is set at 64, the dropout is 0.2, the number of CNN layer is 1, the size of CNN output channel is 256, the number of LSTM layer is 1, the size of LSTM output channel is 128, and 150 epochs of training are performed.

**Table 2:** Network structure and training hyperparameters tuning.

	**Learning rate**	**Epoch number**	**Batch size**	**Dropout**	**CNN layer**	**CNN output channel size**	**LSTM layer**	**LSTM output channel size**
		100						
Range	1e-4 1e-3 1e-2	150 200 250	64 128 256	0.1 0.2 0.3	1,2 3,4	64,128 256,512	1,2 3,4	64,128 256,512
Value	1e-4	150	64	0.2	1	256	1	128

**Figure 4:**
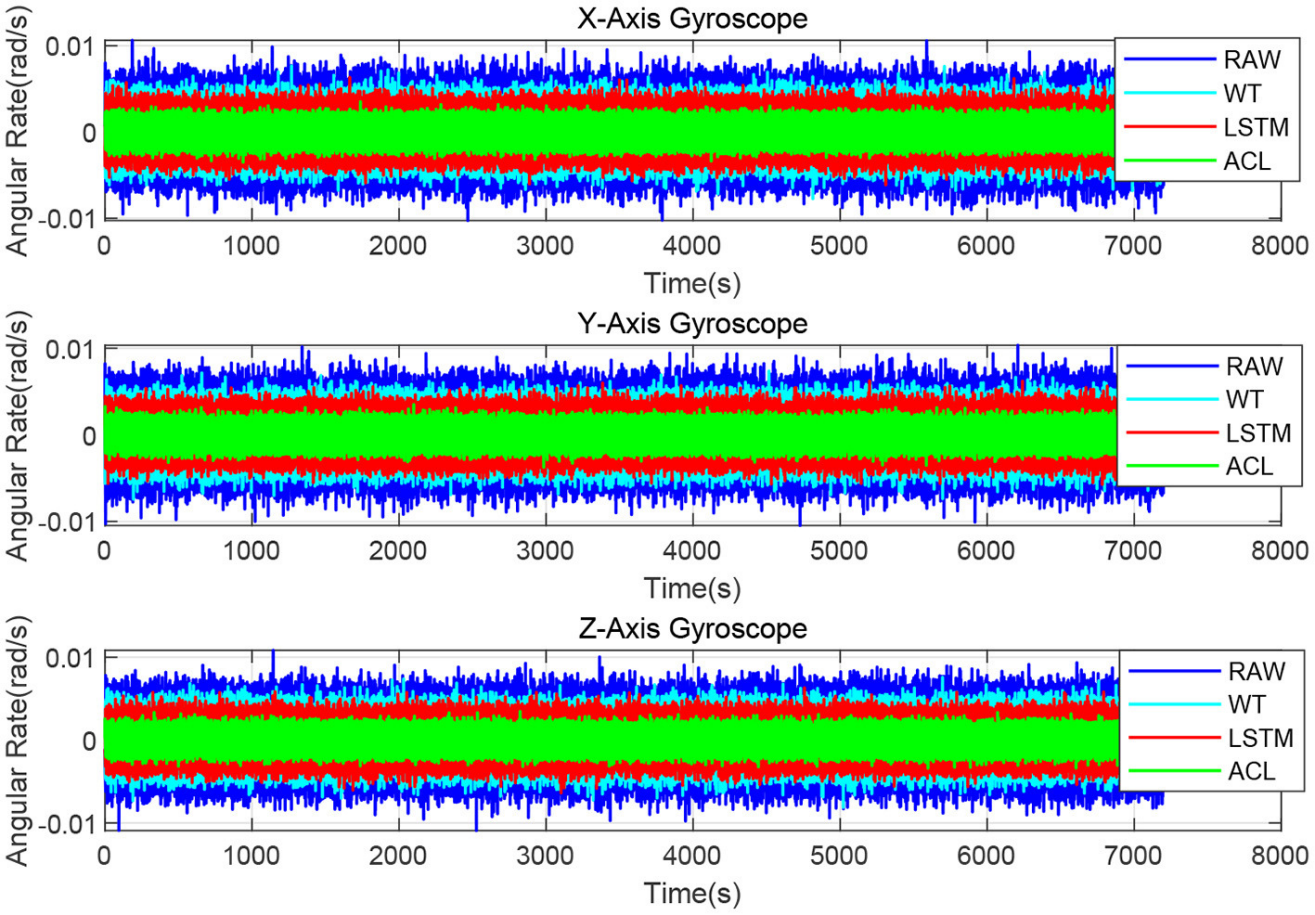
The denoised and raw signals comparison for the 3-axis gyroscope of AHRS380SA.

**Figure 5:**
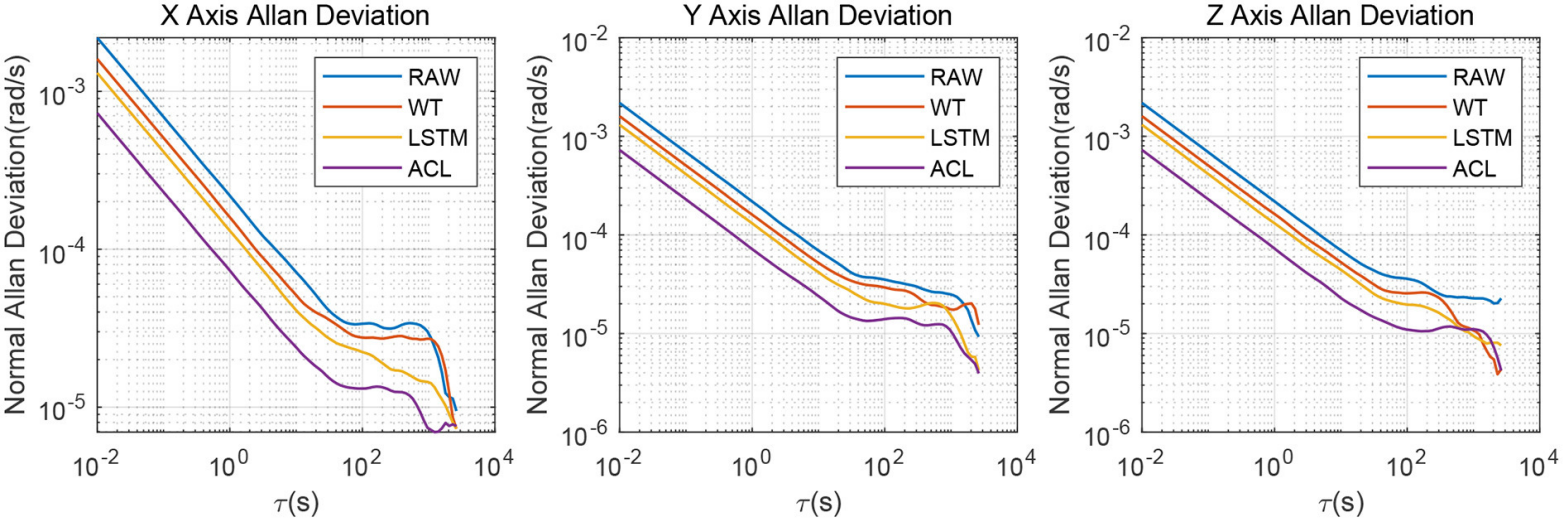
Allan variance comparison between denoised and raw signals for the 3-axis gyroscope of AHRS380SA.

As illustrated in Figure 4, the X/Y/Z-axis raw data and the denoised data of WT, LSTM, and ACL methods are compared in blue, cyan, red, and green curves. LSTM and ACL can achieve significant noise reduction results for static signals better than traditional WT method, and the proposed ACL method has a better denoising effect than LSTM. The root means square error of them are 0.0022, 0.0016, 0.0013, and 0.00073 *rad*/*s*, respectively. Further, the Allan Variance curves comparison results are presented in Figure 5 and the specific error parameters are summarized in Table 3 to distinguish the differences between them. The results show that the ACL method performs the best noise reduction. Especially, the X-axis gyroscope has an improvement of 45.6 and 40.0% in bias instability and angle random walk using the LSTM neural network, while 58.3 and 66.6% using the ACL model. For the Y-axis gyroscope, the bias instability and angle random walk have a 31.3 and 40.0% improvement by the LSTM method, and 53.8 and 66.6% with the ACL model, respectively. For the Z-axis gyroscope, the error of bias instability and angle random walk are decreased by 34.4 and 39.2% using the LSTM method; meanwhile, the ACL model with 58.9 and 64.9%. Thus, according to the analysis of the static experiment, the proposed ACL method has good capability to restrain the stochastic error of the low-cost MEMS gyroscope compared with the application of the WT and LSTM neural network.

**Table 3:** Allan variance parameters of the AHRS380SA 3-axis gyroscope.

**Error sources**		**X-axis**				**Y-axis**				**Z-axis**		

	**Raw**	**WT**	**LSTM**	**ACL**	**Raw**	**WT**	**LSTM**	**ACL**	**Raw**	**WT**	**LSTM**	**ACL**
Bias instability (*deg*/*h*)	9.77	8.76	5.31	4.07	8.14	6.05	5.59	3.76	8.06	6.96	5.29	3.31
Angle random walk ($deg/\sqrt{h}$)	0.75	0.55	0.45	0.25	0.75	0.55	0.45	0.25	0.74	0.56	0.45	0.26

We further test the denoising performances of the proposed method in the dynamic condition. The AHRS380SA IMU is fixed on a turntable, the three axes of which are aligned with the three axes of the turntable. We set the turntable around the Z-axis as the Equation (11), and the sampling frequency is 100 Hz.


(11) $$\begin{array}{c} \omega =2*\sin\left(\pi t/500\right) \end{array}$$

where ω is the angular velocity of the turntable.

Since Allan variance is generally used for static gyroscope data error analysis, root means square error (RMSE) is adopted as an accuracy evaluation index in dynamic experiments, which can reflect the distance between the denoised values and the actual ones. The smaller RMSE, the better the denoising effect, calculated as,


(12) $$\begin{array}{c} RMSE\left(ŷ,y\right)=\sqrt{\frac{1}{m}\overset{m}{\underset{i=1}{\sum }}{\left({ŷ}_{i}-y_{i}\right)}^{2}} \end{array}$$

where *y* is the actual value and ŷ is the denoised value.

**Figure 6:**
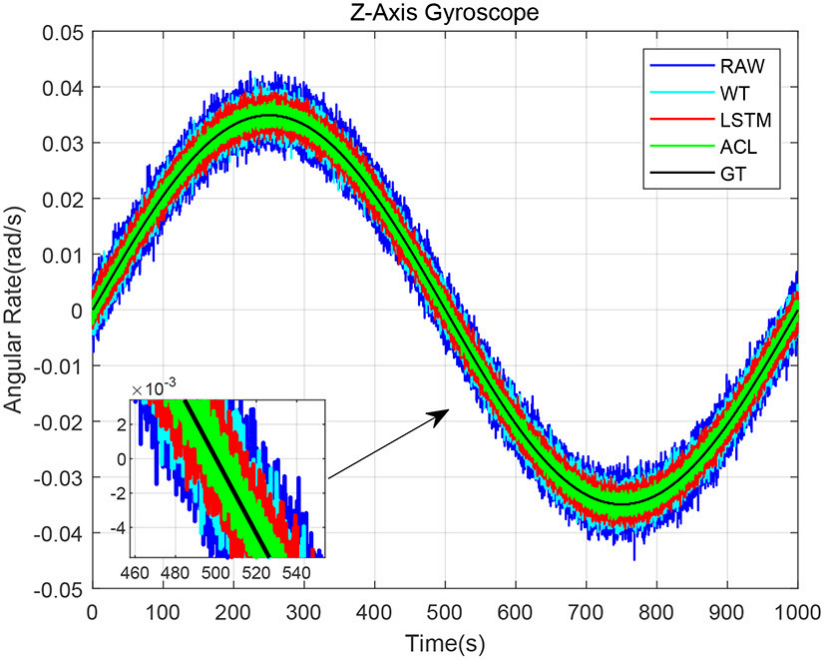
The results of dynamic tests for the AHRS380SA.

Figure 6 shows that three solutions have different effects on the z-axis gyroscope dynamic results. It can be seen that when the turntable angular velocity changes according to our previous setting value, the three denoising methods can track it well. Significantly, the ACL curve in green is closer to the ground truth (GT) curve in black than the LSTM curve in red by magnifying the period from 460 to 540 s. The raw data has the largest RMSE, i.e., 0.0022 *rad*/*s*. The WT and LSTM methods are better than the raw data results, and the RMSE are 0.0016 and 0.0013 *rad*/*s*, respectively. The ACL model has the best performance, the RMSE of which is 0.0007 *rad*/*s*.

### 4.2. Dataset tests

In order to further validate the proposed method, we conducted an open dataset test. Three MEMS IMU datasets with different accuracy in the famous kalibr−allan toolbox (Kalibr-Allan, 2017) are provided by the University of Delaware, i.e., XSENS MTI-G-700, Tango Yellowstone Tablet and ASL-ETH VI-Sensor.

**Figure 7:**
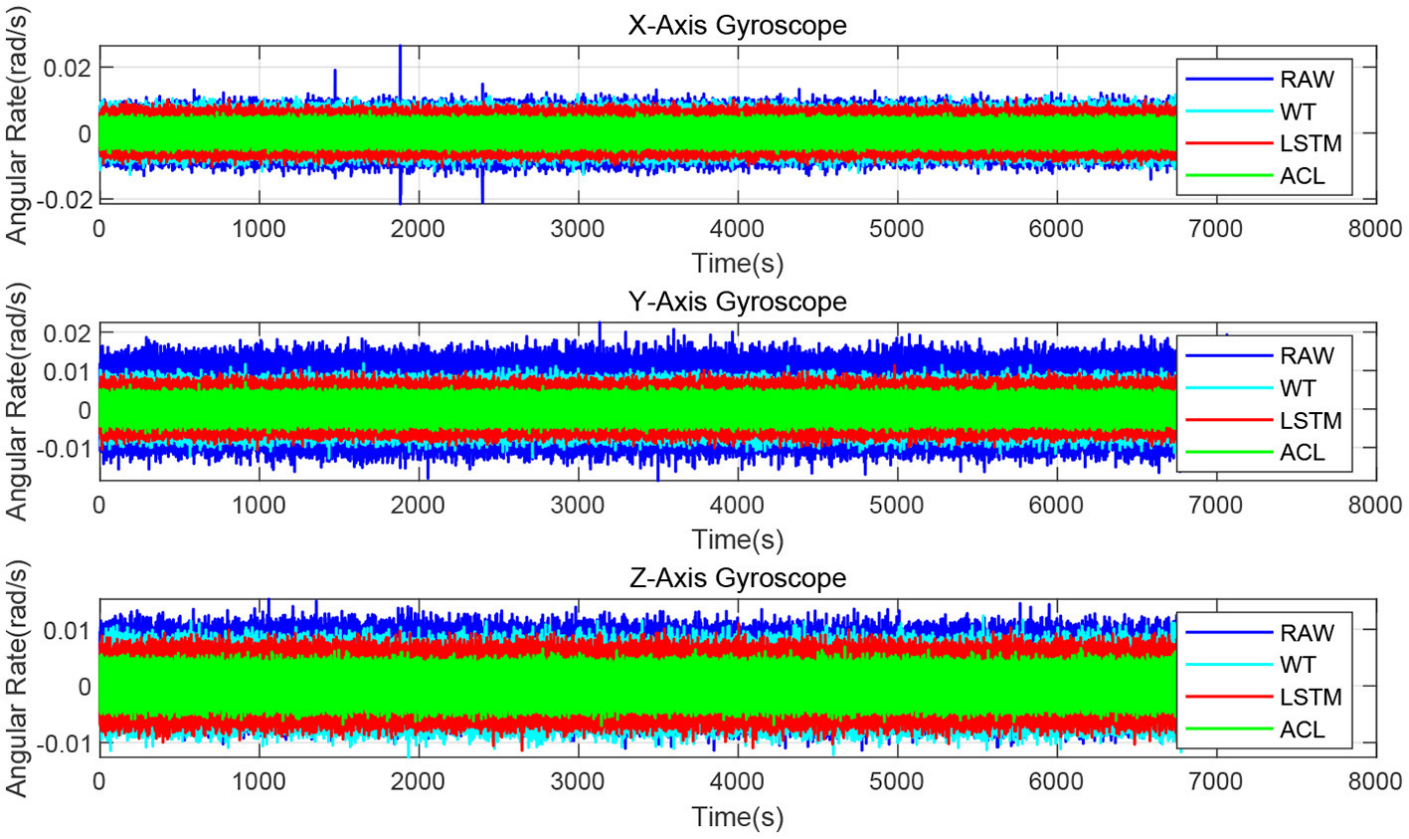
The denoised and raw signals comparison for the 3-axis gyroscope of XSENS MTI-G-700.

**Figure 8:**
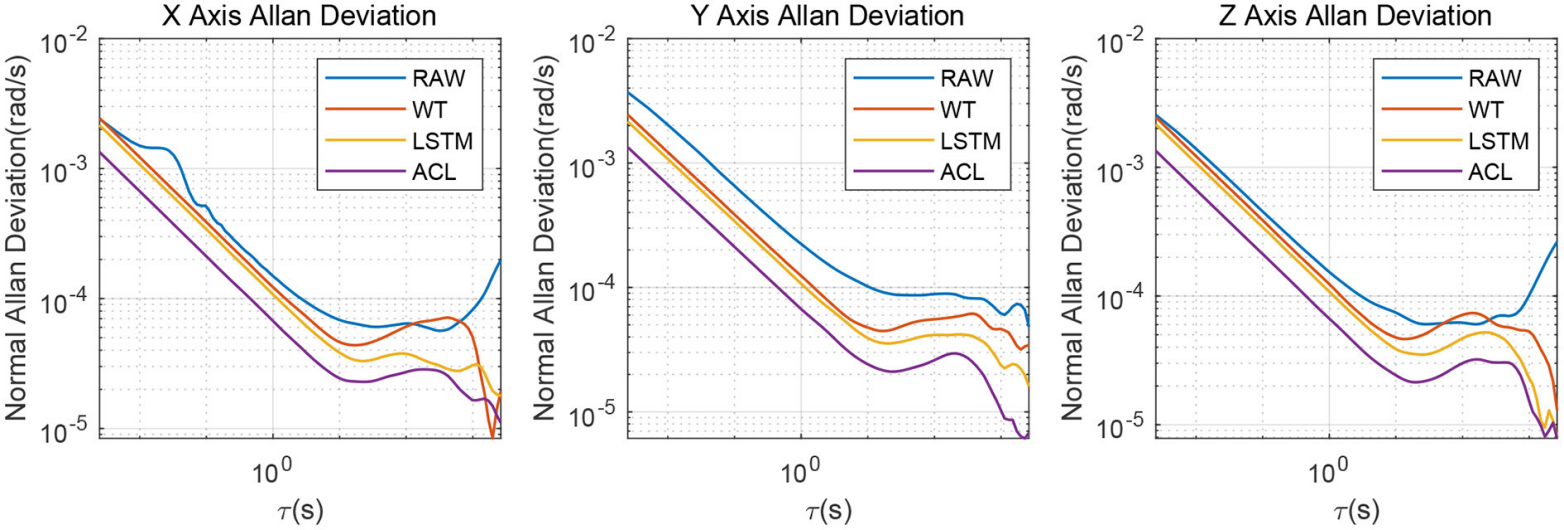
Allan variance comparison between denoised and raw signals for the 3-axis gyroscope of XSENS MTI-G-700.

Since the XSENS MTI-G-700 is a classic low-cost MEMS IMU in inertial navigation, we chose it as our test IMU. The XSENS MTI-G-700 dataset is continuously collected for 3 h at 400 Hz. Similar to real field tests, the results of raw, WT, LSTM and ACL methods are compared in Figure 7. These three noise reduction methods can reduce the peak and peak value of raw data to a certain extent, among which ACL is the best, LSTM is the second, and WT is the worst. The Allan variance curves comparison results are also depicted in Figure 8 and summarized in Table 4: (1) the raw data have the largest average bias instability, i.e., 22.4 *deg*/*h*; (2) the WT is better than the raw data, and the bias instability is reduced to 13.98 *rad*/*s*; (3) the LSTM has an 11.84 *rad*/*s* bias instability; (4) the ACL method performs best in the three solutions.

**Table 4:** Allan variance parameters of the XSENS MTI-G-700 3-axis gyroscope.

**Error sources**		**X-axis**				**Y-axis**				**Z-axis**		

	**Raw**	**WT**	**LSTM**	**ACL**	**Raw**	**WT**	**LSTM**	**ACL**	**Raw**	**WT**	**LSTM**	**ACL**
Bias instability (*deg*/*h*)	20.11	13.62	11.73	7.10	25.30	14.92	12.89	8.14	21.9	13.39	10.90	7.23
Angle random walk ($deg/\sqrt{h}$)	0.51	0.42	0.37	0.23	0.71	0.43	0.38	0.29	0.49	0.42	0.37	0.26

**Figure 9:**
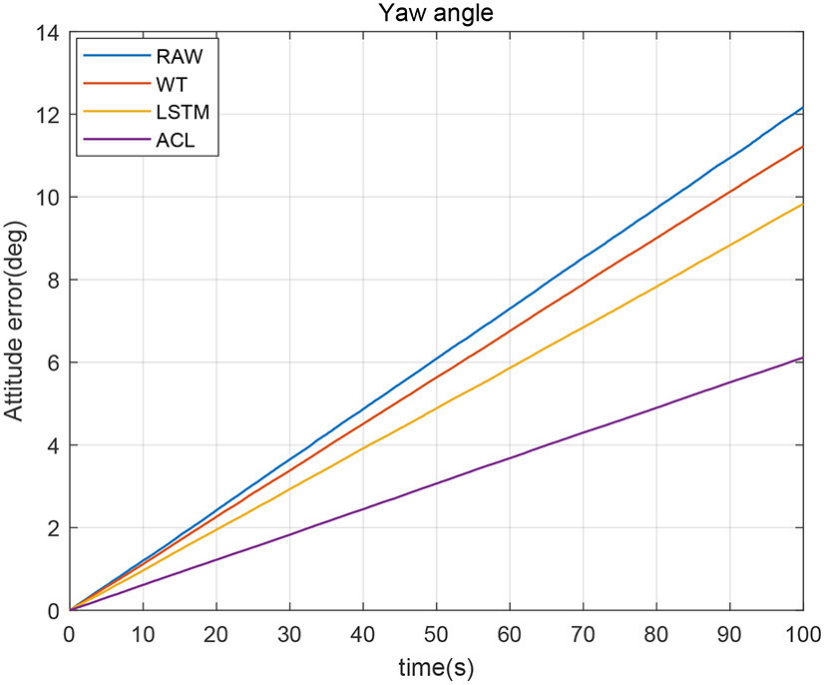
The yaw angle error of the denoised and raw signals.

In order to further analyze the influence of stochastic error on the inertial navigation, we compared the denoised yaw angle errors in Figure 9. The yaw angle error gradually increased with time, and the maximum accumulation error reached 12.2 degrees after 100 s. If the error is not corrected, such a large yaw error cannot be used for inertial navigation. Compared with the yaw angle error of raw data, the denoised yaw angle error divergence over time is effectively improved, where the ACL method basically controls the maximum yaw accumulation error within 6 degrees. The WT and LSTM methods also reduce the degree of yaw angle divergence.

### 4.3. Ablation study

We evaluate the stochastic noise eliminating performance of removing the 1D-CNN and attention mechanism from the ACL model to demonstrate the effectiveness of the proposed ACL design choice of the 1D-CNN and attention mechanism. The ablation study is consist of the AHRS380SA denoised performance comparison between the LSTM, CONV-LSTM, and ACL methods.

**Figure 10:**
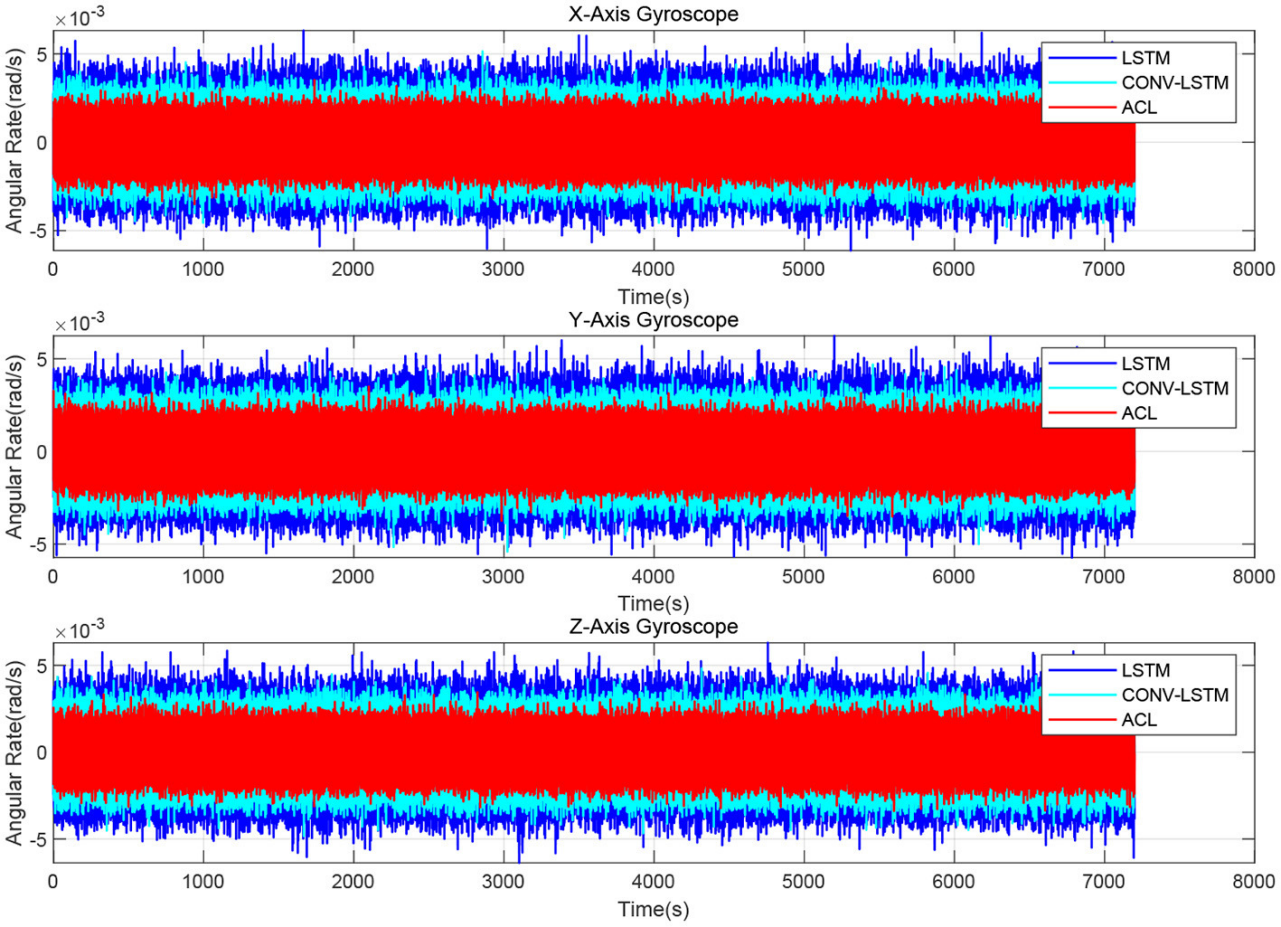
The denoised and raw signals comparison for the ablation study.

**Figure 11:**
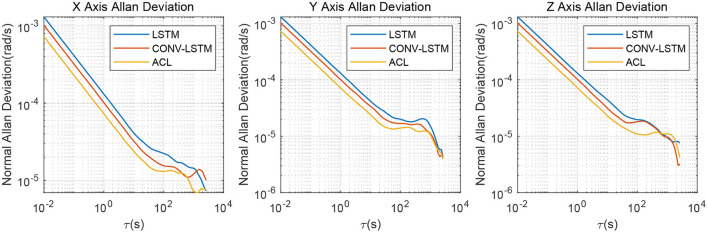
Allan variance comparison between denoised and raw signals for the ablation study.

The denoised results for the AHRS380SA of all the ablation experiments are shown in Figures 10, 11. The average bias instability of the LSTM, CONV-LSTM and ACL are 5.40, 4.90, and 3.71 *deg*/*h*, meanwhile, the average angle random walks are 0.45, 0.32, and 0.24 $deg/\sqrt{h}$, respectively. Applying 1D CNN and attention mechanism in the ACL model has a lower stochastic error than without any attention mechanism after the LSTM layers. The ablation experiments show that all components in the ACL are effective.

## 5. Conclusion

This paper proposes a hybrid denoising method based on deep learning to reduce stochastic errors. The devised deep neural network architecture can predict the gyroscope measurements from various noises. Furthermore, the model combines 1D-CNN and LSTM to extract the local feature representation from the input multivariable time sequences and uses LSTM to correlate the current inputs and historical model information automatically. The attention mechanism is exploited to calculate the weight to improve computing efficiency. In order to verify the performance of the proposed method, numerical real field, dataset and ablation experiments have been performed. Comparing our algorithm with known work in this field, the evaluation results show that our model has greater denoising performances. However, there is still room for improvement, and further research can focus on improving the real-time capability. Furthermore, optimal deep learning based approaches (Reddy et al., 2018) and quantum recurrent network (Gandhi et al., 2013) will be explored for denoising gyroscope in future.

## Data availability statement

The original contributions presented in the study are included in the article/supplementary material, further inquiries can be directed to the corresponding authors.

## Author contributions

YL designed the experiments and algorithms and wrote the main draft of the manuscript. JC and WL participated in the discussion and guided the paper writing. All authors listed have made a substantial, direct, and intellectual contribution to the work and approved it for publication.
